# Targeted Patching and Dendritic Ca^2+^ Imaging in Nonhuman Primate Brain *in vivo*

**DOI:** 10.1038/s41598-017-03105-0

**Published:** 2017-06-06

**Authors:** Ran Ding, Xiang Liao, Jingcheng Li, Jianxiong Zhang, Meng Wang, Yu Guang, Han Qin, Xingyi Li, Kuan Zhang, Shanshan Liang, Jiangheng Guan, Jia Lou, Hongbo Jia, Bingbo Chen, Hui Shen, Xiaowei Chen

**Affiliations:** 1 School of Biomedical Engineering, Tianjin Medical University, Tianjin, China; 2 Brain Research Center, Third Military Medical University, Chongqing, China; 3 Brain Research Instrument Innovation Center, Suzhou Institute of Biomedical Engineering and Technology, Chinese Academy of Sciences, Suzhou, China; 4 Laboratory Animal Center, Third Military Medical University, Chongqing, China; 5 CAS Center for Excellence in Brain Science and Intelligence Technology, Shanghai Institutes for Biological Sciences, Chinese Academy of Sciences, Shanghai, China

## Abstract

Nonhuman primates provide an important model not only for understanding human brain but also for translational research in neurological and psychiatric disorders. However, many high-resolution techniques for recording neural activity *in vivo* that were initially established for rodents have not been yet applied to the nonhuman primate brain. Here, we introduce a combination of two-photon targeted patching and dendritic Ca^2+^ imaging to the neocortex of adult common marmoset, an invaluable primate model for neuroscience research. Using targeted patching, we show both spontaneous and sensory-evoked intracellular dynamics of visually identified neurons in the marmoset cortex. Using two-photon Ca^2+^ imaging and intracellular pharmacological manipulation, we report both action-potential-associated global and synaptically-evoked NMDA (N-methyl-D-aspartate) receptor-mediated local Ca^2+^ signals in dendrites and spines of the superficial-layer cortical neurons. Therefore, we demonstrate the presence of synaptic Ca^2+^ signals in neuronal dendrites in living nonhuman primates. This work represents a proof-of-principle for exploring the primate brain functions *in vivo* by monitoring neural activity and morphology at a subcellular resolution.

## Introduction

The use of nonhuman primates along with rodents in neuroscience research is pivotal for understanding human brain and diseases. Two-photon Ca^2+^ imaging together with patch-clamp recordings allows for elucidating the brain functions *in vivo* at multiple spatial scales ranging from neural populations to individual synapses^1–3^. However, these sophisticated techniques for high-resolution mapping of brain structures and activities were initially developed for rodents and have not been fully available for primate brain research. Recently, *in vivo* two-photon imaging of neuronal activities at the population level with a single-cell resolution has been applied to the monkey neocortex^4–7^, but imaging activities of single synapses, particularly those represent synaptic input-related signals^1, 3, 8^, still remains a major challenge in nonhuman primates.

Neuronal dendrites compute both electrical and chemical signals, a process that is essential for information processing and communication in the brain. One of the most commonly used approaches for analyzing the dendritic signals is to image the dynamics of intracellular Ca^2+^ concentration^3, 9^. Rapid advances in two-photon microscopy and improved fluorescent Ca^2+^ indicators have enabled us to study the dendritic signals under both *in vitro* ^10, 11^ and *in vivo* conditions^1, 12–15^. Using such approaches, several studies have identified sensory-evoked local dendritic Ca^2+^ signals in different sensory cortex regions (visual, auditory, and barrel cortices) in mice and revealed a heterogeneous distribution of sensory inputs on neuronal dendrites^1, 3, 14, 16^. In contrast, recent two-photon imaging work has also identified the functional clustering of synaptic inputs in ferret visual cortex^17^. However, the knowledge of dendritic organization of sensory inputs in the primate brain neurons remains less clear. An essential step forward in addressing this question is to establish a proper technical approach to resolve subthreshold sensory-evoked input signals in the dendrites of primates *in vivo*. Alternatively, one can also apply the techniques that have been already developed in rodents to the primate brain.

In rodent brains, the successful implementation of *in vivo* imaging of neural activity in single dendritic spines, small membranous protrusions in dendrites that correspond to individual afferent excitatory synapses, has benefited from the use of high-speed two-photon imaging termed low power temporal oversampling (LOTOS) procedure^1, 18, 19^ or the use of a new generation of genetically encoded Ca^2+^ indicators (GCaMP6s)^15^. Alternatively, conventional two-photon imaging has also been used for *in vivo* spine Ca^2+^ imaging, but this was only achieved when the membrane potentials of the recorded neurons were strongly depolarized to −30 mV to 0 mV to maximize the NMDA receptor-dependent Ca^2+^ responses^20^. In monkey cortex, *in vivo* imaging of dendritic spines has been restricted to the study of their morphology and turnover^7^. In the present study, to image the activities of dendrites and spines in primate brain, we chose to use the LOTOS procedure in conjunction with whole-cell patch-clamp recordings in the adult marmoset neocortex. Moreover, we chose to use a synthetic Ca^2+^ dye, as it offers a practical advantage over genetically encoded Ca^2+^ indicators, namely an easy-to-deliver property through the patch electrode^2^. As a result, we were able to simultaneously monitor intracellular electrical signals from the soma and Ca^2+^ signals from the dendrites of visually identified neurons in the marmoset brain *in vivo*.

## Materials and Methods

### Animals

Two male common marmosets (*Callithrix jacchus*; 58 and 60 months old; body weight: 290 g and 300 g, respectively) were provided by the Laboratory Animal Center at the Third Military Medical University. The colony room is maintained at a temperature range of 27 °C–29 °C and a relative humidity of 50–60%, with a 12 L:12D light cycle. Marmosets live in family groups. They were fed with fruits and vegetables, accessed to water ad libitum. In the experiment day, peanuts and dried fruits were used to attract them to move to a transfer cage or an anesthesia cage. The marmosets were not used for other experiments before surgery and anesthesia. All experimental procedures were performed in accordance with institutional animal welfare guidelines and were approved by the Third Military Medical University Animal Care and Use Committee.

### Surgery

**Figure 1:**
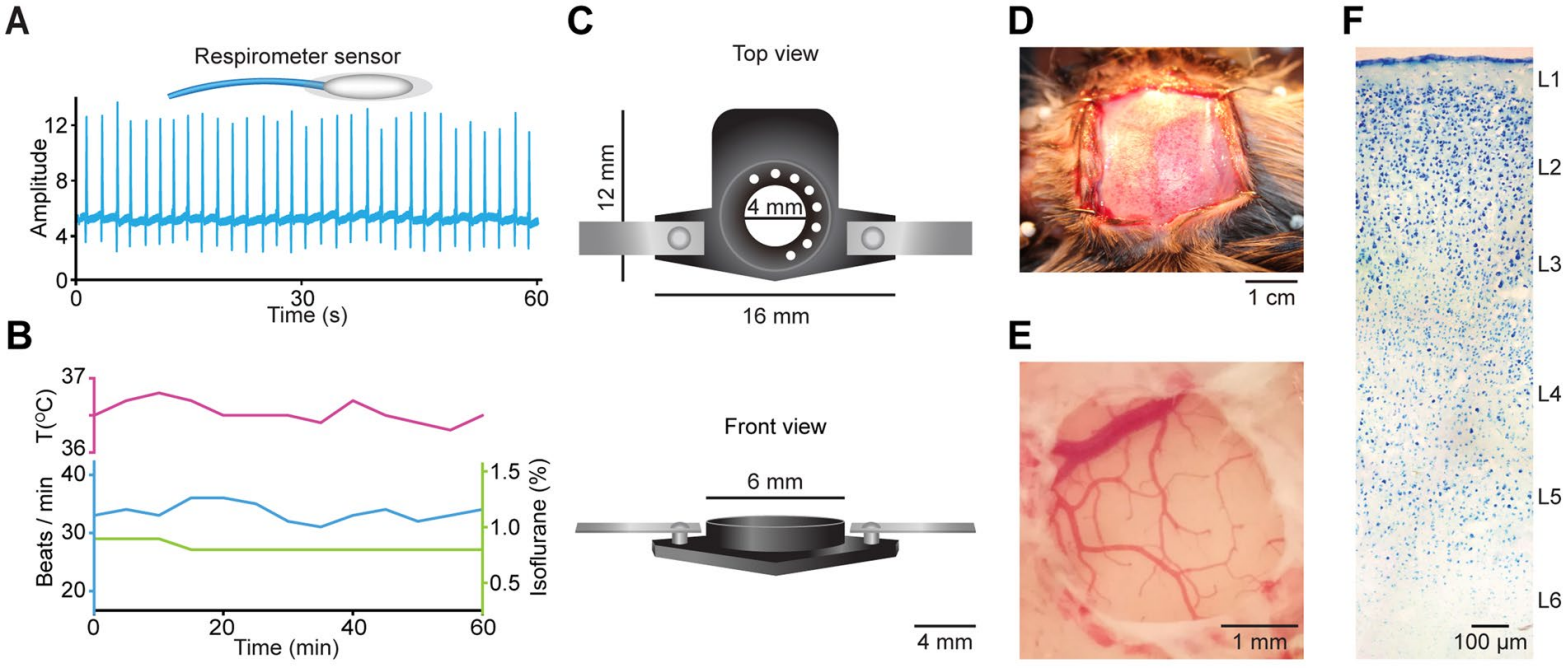
The Marmoset’s physical condition and preparation for two-photon Ca^2+^ imaging in marmoset cortex *in vivo*. (**A**) Respiration rate recording. Upper, Cartoon of the sensor of respirometer. Lower, respiration activity over a 60 sec recording period. **(B)** Body temperature (upper, purple), breathing rate (middle, blue) and isoflurane concentration (lower, green) over a 60 min recording period. **(C)** Cartoon of the custom-made chamber (black part: length 16 mm, width 12 mm, innerdiameter 4 mm; outer diameter 6 mm; height 5 mm) and holder (grey part, length 10 mm for one side). Upper, top view. Lower, front view. (**D**) Exposed skull. (**E**) Exposed primary somatosensory cortex. (**F**) Nissl staining of the marmoset neocortex, showing all 6 layers (L).

Marmosets were anesthetized by 1.5% isoflurane with pure oxygen, and kept in a stereotaxic apparatus with a heating pad setting to 38–39 °C in a prone position. We monitored the anesthesia state independently and the level of anesthesia was confirmed by the absence of tail-pinch or paw reflexes. The whole procedure of anesthesia was performed by a clinical certified anesthetist. The concentration of isoflurane was stable (Fig. 1) and was adjusted slightly and carefully only in accordance with the state of the animal. The surgical procedures were performed under aseptic environment. Ampicillin (40 mg kg^−1^) and carprofen (5 mg kg^−1^) were injected intramuscularly. Rectal temperature and breathing rate were monitored throughout the procedure (Fig. 1A,B). The fur and skin over parietal skull were removed after local lidocaine injection (2%). A custom-made plastic chamber was then fixed to the skull with cyanoacrylic glue (UHU) and dental cement (Tetric EvoFlow from Ivoclar Vivadent Corporate) over the left primary somatosensory cortex according to stereotaxic coordinates^21, 22^ (Fig. 1C–E). We used a cranial drill to make a circular craniotomy (~3 mm diameter). The dura mater was removed by a forceps and the exposed cortex was covered with 1.5% low-melting-point agarose. Afterwards, the marmoset was transferred and fixed on the recording setup. Unfortunately, one marmoset died around one hour after we transferred it to the recording setup, which was probably due to a high level of isoflurane (1.2–1.5%). Therefore, for another animal, we reduced the level to 0.6–0.8% throughout the entire experiments. In this condition, the breathing rate was stabilized in the range of 30–40 breaths per min (Fig. 1A,B). The recording chamber was perfused with normal artificial cerebral spinal fluid (ACSF) containing 125 mM NaCl, 4.5 mM KCl, 26 mM NaHCO_3_, 1.25 mM NaH_2_PO_4_, 2 mM CaCl_2_, 1 mM MgCl_2_ and 20 mM glucose (pH 7.4 when bubbled with 95% oxygen and 5% CO_2_). The rectal temperature of marmoset was kept between 36.5–37.5 °C throughout the experiments (Fig. 1B).

### Two-Photon Ca^2+^ Imaging

Two-photon imaging was performed with a custom-built two-photon microscope system based on 12 kHz resonant scanner (model “LotosScan 1.0”, Suzhou Institute of Biomedical Engineering and Technology), similar to that described previously^14, 16^ (Jia *et al*.^14^; Varga *et al*.^16^). Two-photon excitation light was delivered by a mode-locked Ti:Sa laser (model “Mai-Tai DeepSee”, Spectra Physics), and a 40 × /0.8 NA water-immersion objective (Nikon) was used for imaging. For Ca^2+^ imaging experiments, the excitation wavelength was set to 920 nm. For somatic and dendritic Ca^2+^ imaging, we acquired images of 600 × 600 pixels at 40 Hz frame rate. The size of field-of-view was ~ 200 µm × 200 µm. The average power delivered to the brain ranged from 30 to 80 mW, depending on the depth of imaging. For spine Ca^2+^ imaging, we changed the imaging system to the LOTOS scanning mode^1, 15, 16, 18^. We reduced the number of lines to 64 and the number of pixels in each line to 256. The imaging repetition rate was 200 Hz. The width of the field-of-view was therefore reduced to 27–42 µm. The average power delivered to the brain was 15–30 mW. Within an imaging time window of ~5 min for each dendritic field, no sign of photodamage was observed.

### Hind-limb Electrical Stimulation

For hind-limb electrical stimulation, electrical stimulation (duration 1 s, intensity 0.1–0.6 mA) was delivered to the hind-paws through two 30-gauge needles connected to an isolated pulse stimulator (Isolated Pulse Stimulator Model 2100, AM-system).

### Whole-Cell Patch-Clamp Recordings

**Figure 2:**
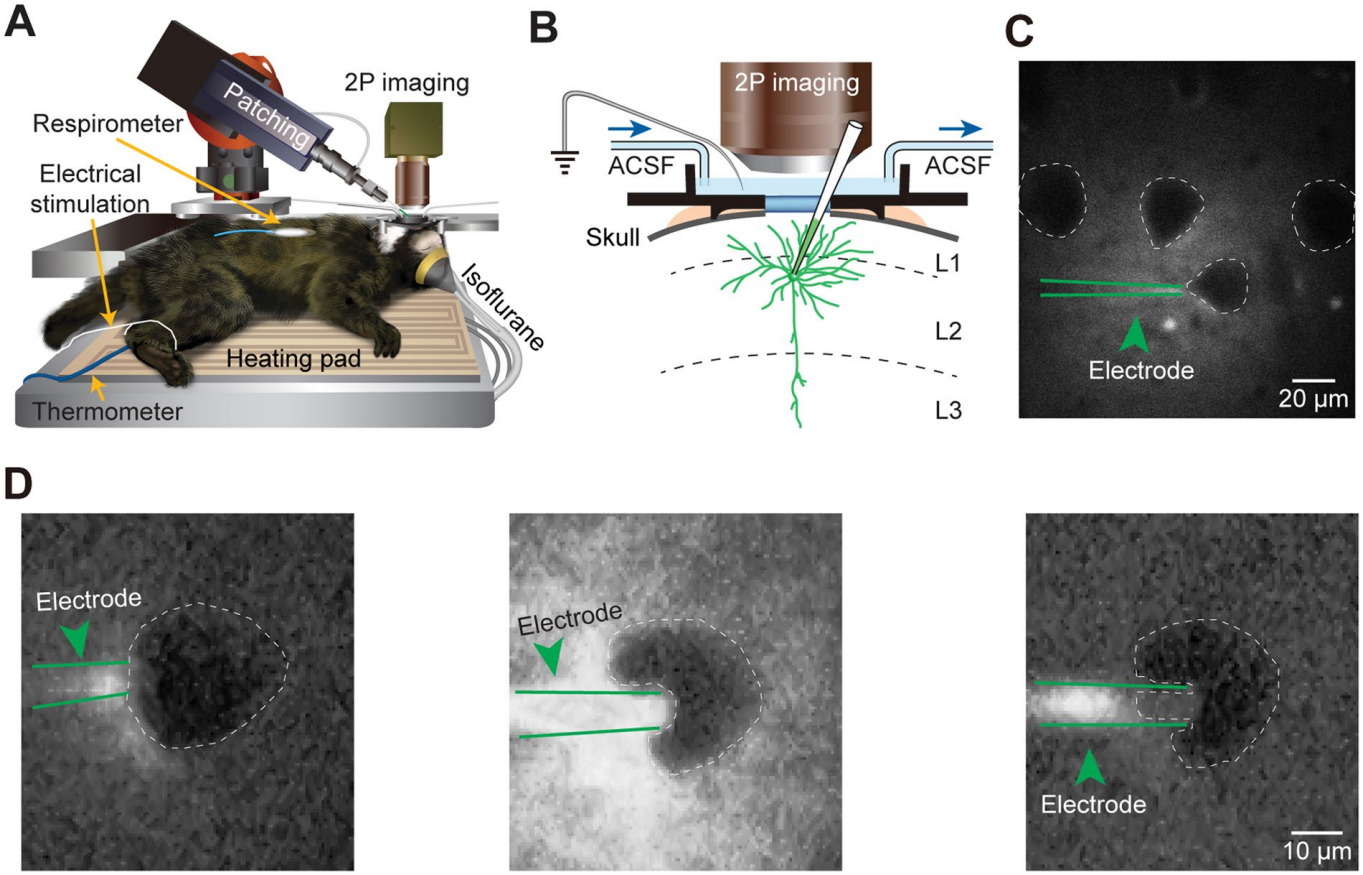
Two-photon targeted whole-cell recordings in marmoset cortical neurons *in vivo*. (**A**) Schematic of two-photon imaging and whole-cell recording in a marmoset anesthetized with isoflurane. The yellow arrows point to the respirometer sensor, the thermometer and the wire of electrical stimulation. (**B**) Side view of the experimental arrangement, showing the patch electrode filled with OGB-1, the water-immersion objective, the agarose-covered cortical surface, the recording chamber, and the flow direction of ACSF perfusion into the cortical surface. **(C)** OGB-1 was blown out from an electrode and the neurons can be identified as shadows which were delineated with white dotted contours. The electrode is pointed by a green arrowhead and delineated with green lines. (**D**) The process of obtaining whole-cell configuration. Left, the electrode was approaching the shadow. Middle, the neuron was pressed by the pipette positive pressure. Right, the pressure was released and suction was applied to the pipette to form a gigaohm seal.

For whole-cell patch-clamp recordings in marmoset primary somatosensory cortex neurons *in vivo*, we used the”shadowpatching” procedure according to the previous studies^1, 23^. Recordings were done with an EPC10 amplifier (USB double, HEKA Elektronik). Electrophysiological data were filtered at 10 kHz and sampled at 20 kHz using Patchmaster software (HEKA). The pipette electrodes pulled from borosilicate glass capillaries (BF150–86–10, Sutter Instrument Company, Novato, CA) had a resistance of 4–6 MΩ when filled with internal solution containing 112 mM potassium gluconate, 8 mM KCl, 10 mM HEPES, 4 mM Mg-ATP, 0.375 mM Na_2_GTP, 10 mM sodium phosphocreatine and OGB-1 potassium salt (200 μM; Invitrogen). The patch pipette penetrated the pia mater with positive pressure of 100–200 mbar and was then reduced to ~ 20–30 mbar to obtain good images of both electrode and shadow neurons under two-photon imaging (Fig. 2A–C). After identification of the cell of interest, the tip of the pipette electrode pushed against its membrane. When the tip resistance raised 3–5 MΩ, we released positive pressure and suctioned to obtain a gigaohm seal. After gigaohm seal formation, gentle suction was applied to break through the cell membrane to establish whole-cell configuration (Fig. 2D). The recording of the best two cells lasted up to ~2 hours and the recording duration of other eight cells varied from 30 minutes to 1 hour). For intracellular pharmacological manipulation, we included MK-801 (1 mM) in the internal solution. The series resistance was continuously monitored and the data were rejected for analysis if the resistance was higher than 30 MΩ. Dendritic Ca^2+^ imaging was started at least 20 min after formation of whole-cell configuration. The experiments were done in one session lasting17 hours. The state of the animal was stable (body temperature: 36.5 °C; respiration rate: 30–40 breaths per min) and carefully monitored until the end of the recording session.

### Nissl Staining

After imaging and electrophysiological experiments, the marmoset was deeply anesthetized by an intraperitoneal injection of sodium pentobarbital and perfused transcardially with 0.9% NaCl, followed by fixation with 4% paraformaldehyde in 0.1 M PBS (pH 7.4). The animal was carefully euthanized in accordance with institutional animal welfare guidelines, and it was approved by the Third Military Medical University Animal Care and Use Committee. The cryoprotected marmoset brain was sliced with a thickness of 40–50 µm. The slices were rehydrated and stained in 0.1% cresyl violet solution for 3–10 minutes. Then, they were soaked in 95% ethyl alcohol for 5–30 minutes, dehydrated in 100% alcohol and cleared in xylene for 5 minutes. At last, the imaging was obtained with a stereoscope (Olympus) (Fig. 1F).

### Data Analysis

The analyses of electrophysiological and Ca^2+^ imaging data were conducted offline by using custom-written software in LabVIEW 2012 (National Instruments), Igor Pro 5.0 (Wavemetrics) and Matlab 8 (Mathworks). For correcting lateral motion in the imaging data, a rigid-body transformation based frame-by-frame alignment was applied by using Turboreg software (ImageJ plugin). For reconstructing morphology of the recorded neuron, the fluorescent image projections of the single neuron were generated from *z*-stack of two-photon images using Simple Neurite Tracer software (ImageJ, http://rsb.info.nih.gov/ij/), and then the dendrites were manually tracked using Adobe Illustrator CS6 (Adobe Systems).

To extract fluorescence signals from imaging data, regions of interests (ROIs) were visually identified and drawn based on fluorescence intensity. All pixels within each specified ROI were averaged to estimate fluorescence changes (f). Relative fluorescence changes Δf/f = (f-f_0_)/f_0_ were calculated as Ca^2+^ signals, where the baseline fluorescence of the ROI f_0_ was estimated as the 25^th^ percentile of the fluorescence within a sliding time window (window size: 10 s). Regions of interest for spine calcium imaging analyses were restricted to the clearly visible protrusions emanating laterally from the dendritic shaft, as described previously^1, 18, 19^. To remove the noises from respiration (rate = 0.5–0.6 Hz) and heartbeat (rate = 4–5 Hz)^24^, the Δf/f traces shown in all figures were first notch filtered (0.6 and 4 Hz) and then low-pass filtered (0–5 Hz) with Butterworth filter (3rd order).

Summarized data were presented in figures as mean ± standard error of the mean (SEM). To compare data from two groups, we used Wilcoxon rank sum test to determine statistical significance between them. P < 0.05 was considered statistically significant.

## Results

### Intracellular Dynamics of Marmoset Cortical Neurons Revealed by Two-Photon Guided Whole-Cell Recordings

**Figure 3:**
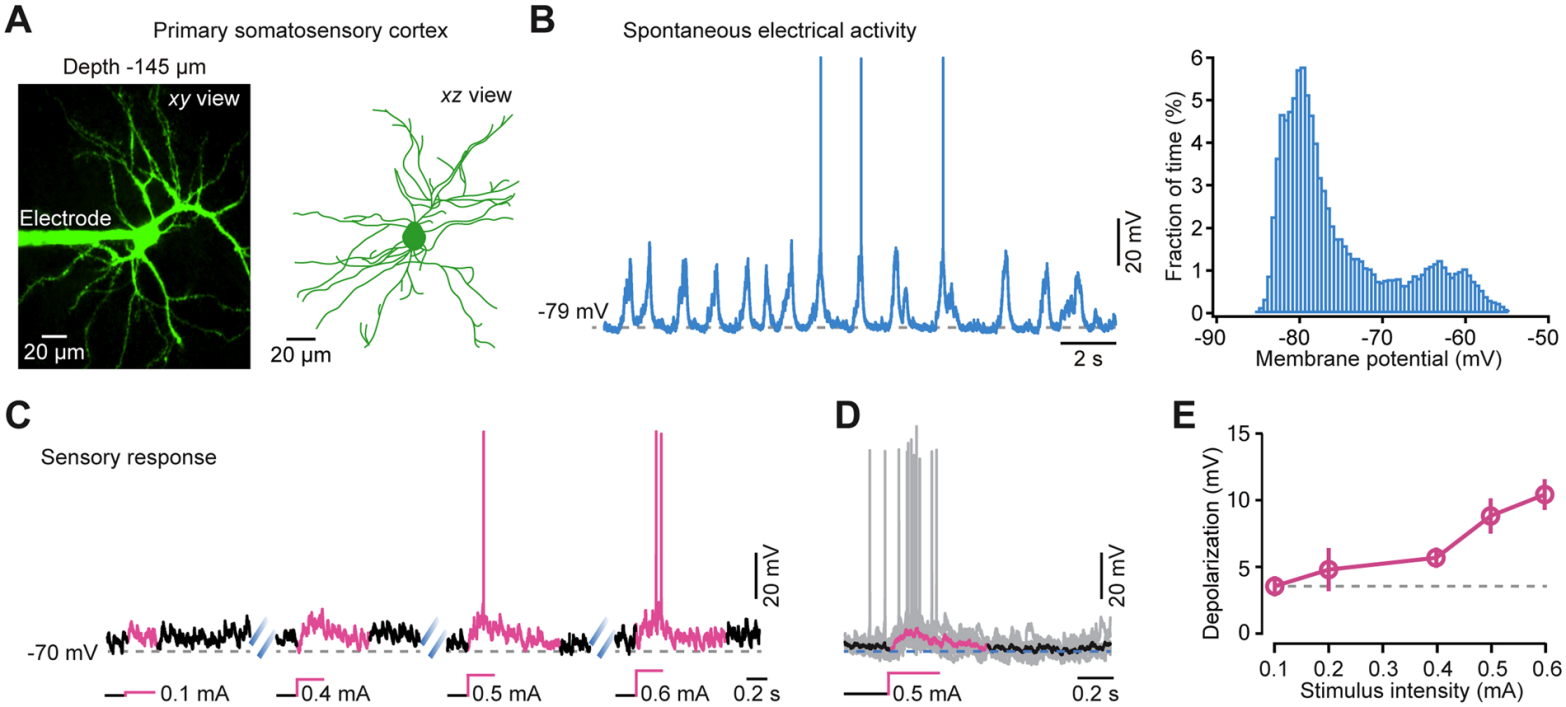
The spontaneous and stimulation-evoked electrical responses in marmoset cortical neurons. (**A**) Z-projection of *in vivo* two-photon images of a whole-cell patched layer 2 (L2) pyramidal cortical neuron. Left, top view of this neuron. Right, side view of the neuron reconstructed from two-photon images. (**B**) Left, representative whole-cell recording of spontaneous activity from the same L2 neuron as in panel (**A**) under anesthesia. Right, distribution of membrane potentials of this neuron during a 45 s recording period. (**C**) Neuronal responses to different intensities of electrical stimulation of the hind-limb from another neuron**. (D**) Overlay of sensory responses to 0.5 mA electrical stimulation from 7 traces. (**E**) Intensity dependence of stimulus-evoked depolarization (*n* = 4).

We performed targeted whole-cell recordings using the “shadowpatching” procedure^1, 23^ in the hind-limb region of the marmoset somatosensory cortex under isoflurane anesthesia (Fig. 2A). By means of two-photon imaging through a cranial window, we patched neurons under direct visual control with a glass electrode containing the Ca^2+^-sensitive fluorescent dye Oregon Green BAPTA-1 (OGB-1). The somata of the patched neurons were located in the superficial layer at a depth of 120–300 µm, corresponding to the neurons within layer 2 (Figs 1F and 2B)^25, 26^. The most evident limitation of using this technique in deep layer neurons is the fact that the pipette would be contaminated more frequently since it needs to penetrate thicker tissues. This would significantly reduce the success rate of obtaining a high-quality patch-clamp recording. The neurons were passively loaded with the Ca^2+^ dye after achieving whole-cell configuration. At the end of recordings of each neuron, the morphology was reconstructed from *z*-stack of two-photon images, revealing a pattern of numerous apical, oblique and basal dendrites (see an example in Fig. 3A). Current-clamp recordings at the neuronal somata revealed slow oscillations of membrane potentials consisting of an alternation of depolarized (UP) and hyperpolarized (DOWN) states (UP state frequency was in the range of 0.52 to 0.76 Hz; 0.65 ± 0.07 Hz, *n* = 7 neurons; Fig. 3B, left panel), showing a bimodal distribution of membrane potentials (Fig. 3B, right panel). The membrane potentials at DOWN states ranged from −80 to −65 mV (−71 ± 2.2 mV, *n* = 7). The membrane potentials were not corrected for liquid junction potentials. Action potential (AP) firing was sparsely found during UP states (0.019 ± 0.004 Hz, *n* = 7, calculated from both UP and DOWN states). These properties are similar to those described for mouse superficial-layer cortical neurons under the same condition^1, 19^. Previous studies using extracellular recordings have suggested similar slow oscillations during slow-wave sleep^27^. In addition, electrical stimulation of the hindlimb produced responses in the recorded neurons. Increasing stimulus intensity resulted in a progressive increase in the amplitude of postsynaptic potential responses and reliably produced spiking when the stimulus was above 0.5 mA (Fig. 3C–E). Thus, we successfully establish targeted whole-cell recordings in the marmoset cortex, allowing for the analysis of intracellular dynamics of single identified neurons in the intact brains of nonhuman primates.

### Action Potential-Associated Ca^2+^ Signals in the Dendrites of Marmoset Cortical Neurons

**Figure 4:**
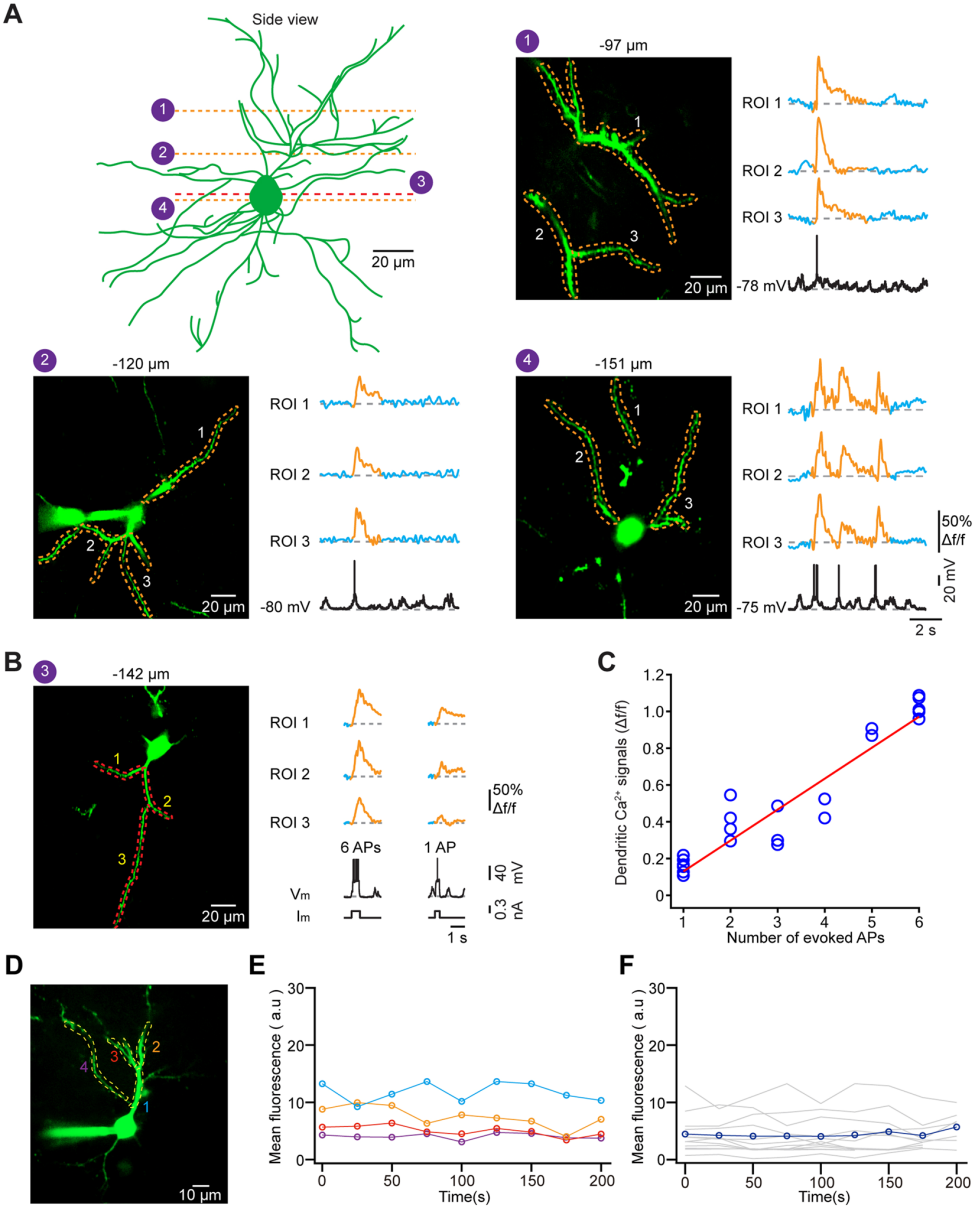
Global dendritic Ca^2+^ signals associated with action potentials. (**A**) Upper left panel, side view of a recorded neuron, the same as in Fig. 3A. Four different planes of focus are indicated by dotted lines. Three other panels, dendritic Ca^2+^ transients associated back-propagation action potentials (APs) in these three planes of this neuron. Each panel: left, two-photon image with regions of interest (ROIs); right, Ca^2+^ transients (Δf/f) from the corresponding ROIs and the somatic electrical activity. (**B**) Ca^2+^ transients from three ROIs during 6 APs and 1 AP that were evoked by current injections. Left, two-photon Ca^2+^ image with ROIs. Right, Ca^2+^ transients from these ROIs, and their corresponding membrane potentials (Vm) and current injections (Im). **(C)** Dependence of amplitude of dendritic Ca^2+^ transients on the number of APs that were evoked by current injections. Each blue circle represents the result from one dendritic region of interest and the red line indicates a linear fit (R^2^ = 0.861, *P* = 0.0048; *n* = 6 neurons) (**D**) Two-photon Ca^2+^ image with four ROIs from another cell. **(E)** Mean fluorescence from these ROIs during a 200 s recording. **(F)** Mean fluorescence from 13 different dendritic regions of three neurons.

In combination of whole-cell recordings, we next performed two-photon imaging to explore back-propagating AP-associated dendritic Ca^2+^ signals in marmoset cortical neurons. We carried out imaging at a speed of 40 frames per second to achieve a resolution at the level of dendrites^14, 16^. Figure 4A,B show an example of such recordings, in which we obtained four different planes of focus from apical to basal dendrites in the same neuron. We observed that APs produced Ca^2+^ transients invading effectively all imaged dendritic branches. Even for single APs, we were able to detect these global dendritic Ca^2+^ signals. These global dendritic signals could be evoked by current injections through the patch electrode (Fig. 4B), indicating that such signals are largely resultant from the activation of voltage-gated Ca^2+^ channels by AP backpropagation, with a possible contribution of coincidently-occurring local synaptic activation, consistent with the results from L2/3 neurons in mouse cortex *in vivo* ^1, 19, 28^. As indicated by quantitative analysis, the amplitude of AP-associated Ca^2+^ transients evoked by current injections showed a significant dependence on the number of APs (Fig. 4C; *n* = 6).

### Subthreshold Ca^2+^ Signals in the Dendrites of Marmoset Cortical Neurons

**Figure 5:**
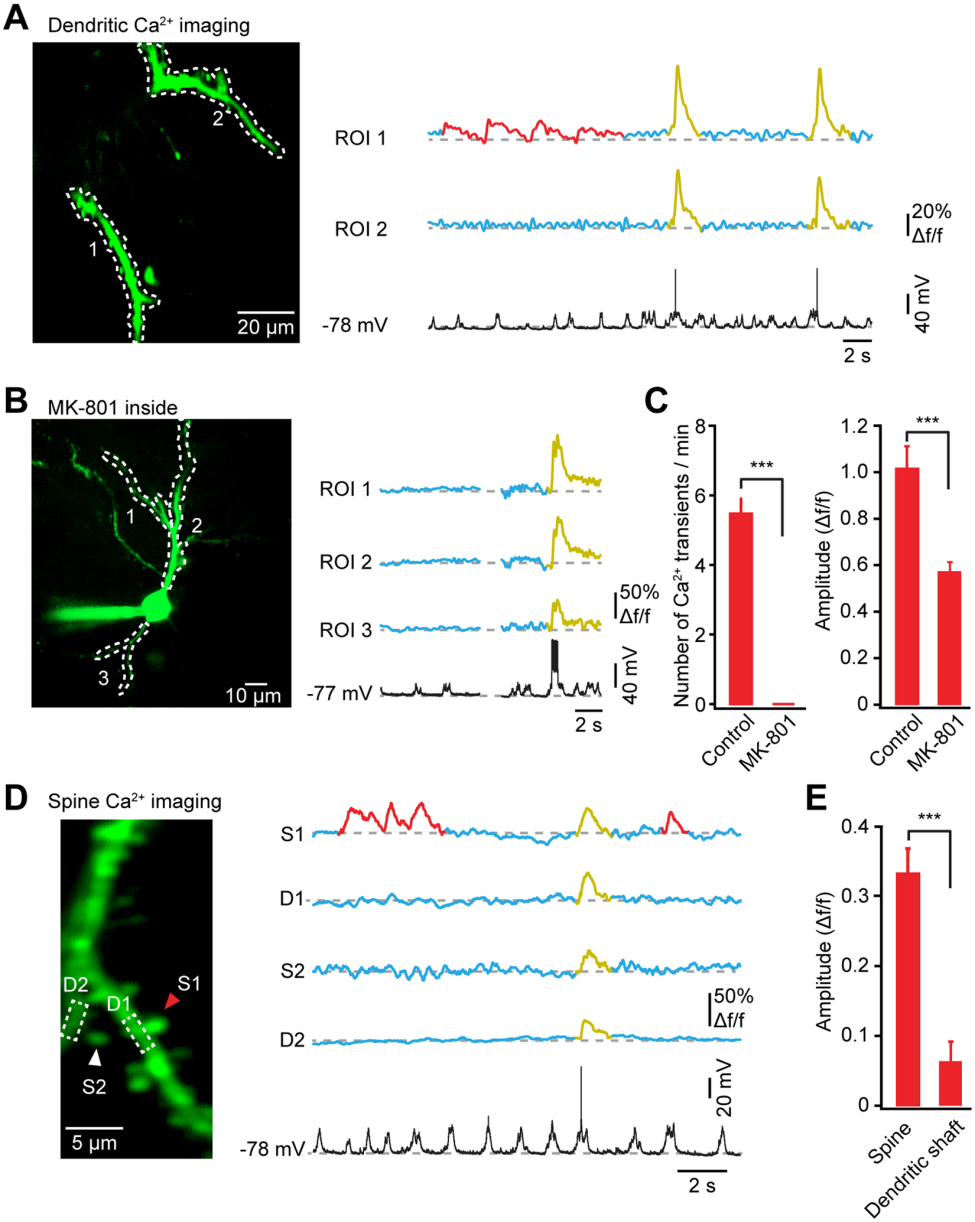
Local Ca^2+^ signals associated with synaptic activation in dendrites and spines. (**A**) Subthreshold local dendritic Ca^2+^ signals. Left, two-photon image with ROIs from the same neuron as in Fig. 3A. Right, local dendritic Ca^2+^ signals (in red) and AP-associated dendritic Ca^2+^ signals (in yellow), and their corresponding electrical signals recorded from the soma (in black). **(B)** Blockade of local dendritic Ca^2+^ signals by intracellularly applied MK-801 (1 mM). Note that AP-associated Ca^2+^ signals were still present, obtained from the same neuron in Fig. 4D. (**C**) Comparison of number of subthreshold dendritic Ca^2+^ transients (Left) and amplitude of single AP-associated dendritic Ca^2+^ transients (right) without (control: *n = *10 dendrites from 5 neurons) and with MK-801 (*n* = 8 dendrites from 3 neurons). Wilcoxon rank sum test, ***P < 0.001. Error bar shows SEM. **(D)** Subthreshold spine Ca^2+^ signals. Left, two-photon image of a dendritic segment, obtained from the same neuron as in Fig. 3A. Right, Ca^2+^ signals from two spines (marked by arrowheads) and their dendritic shafts (outlined by dotted boxes) in this dendritic segment, and the corresponding somatic electrical signals. Local Ca^2+^ signals are marked in red, while AP-associated signals are marked in yellow. **(E)** Summary of the amplitude of local Ca^2+^ transients in spines and in their dendritic shafts (10 spines from 3 neurons). Wilcoxon signed rank test, ***P < 0.001.

In addition to the observation of AP-associated Ca^2+^ transients, in many occasions we also found local dendritic Ca^2+^ signals, probably representing the synaptic input signals through the activation of NMDA receptors^14, 16^. For example, in the plane of focus shown in Fig. 5A, several subthreshold Ca^2+^ transients spontaneously occurred in “dendrite 1” (ROI1), but not in “dendrite 2” (ROI2). In contrast, a single AP produced Ca^2+^ transients in both dendrites. Overall, such local dendritic Ca^2+^ transients were observed in 10 out of 22 dendrites (*n* = 5 neurons), which was less frequent that the results observed in mouse cortical neurons^1, 19^. Across all the imaged dendritic branches that had local Ca^2+^ signals, we found that their frequency was about 5 transients/min (*n* = 10 dendrites). Based on a number of studies performed in mouse cortical neurons both *in vitro* ^28, 29^ and *in vivo* ^1, 14, 16, 30^, the major source of these local dendritic signals is synaptically-driven NMDAR-dependent Ca^2+^ entry. To confirm whether this is also true for marmoset cortical neurons, we intracellularly applied MK-801, a NMDA channel blocker, into the recorded neurons through the patch electrode. We observed that MK-801 completely abolished the local dendritic Ca^2+^ signals. However, the global dendritic Ca^2+^ signals evoked by current injection-induced AP backpropagation were still present, with a reduction in their amplitudes (Fig. 5B,C: right panels). It should be mentioned that MK-801 might affect the local circuit excitability as we moved the electrode through the tissue towards the cell.

### Ca^2+^ Signals in Dendritic Spines of Marmoset Cortical Neurons

For imaging dendritic Ca^2+^ signals at the level of single spines, we switched the imaging system to the LOTOS scanning mode that has a repetition speed of 200 frames/s and needs only a low excitation laser power per frame. Figure 5D shows an example of spine imaging, in which we observed Ca^2+^ transients only in “spine 1” (S1) in the absence of APs, without any detectable signals in the parent dendritic shaft (D1). However, other spines in this dendritic branch, for example “spine 2” (S2), did not show any subthreshold Ca^2+^ signals during the same period. As a control, an AP reliably produced Ca^2+^ transients in all ROIs. Overall, we found local Ca^2+^ signals in 10 spines, with no or smaller signals in their parent dendrites (Fig. 5E). These results demonstrate the recordings of synaptic Ca^2+^ signals in dendritic spines in marmoset cortical neurons.

## Discussion

In this study, we have successfully adapted the technique used for investigating dendritic functions *in vivo* that was initially developed in mice^1, 18^ to study a New World primate, common marmoset. In its cerebral cortex, we have provided a proof-of-principle for the establishment of two major challenging *in vivo* methods, including (1) targeted whole-cell patch-clamp recordings in visually identified neurons, and (2) two-photon Ca^2+^ imaging in neuronal dendrites.

Up to now, it still remains a great technical challenge to perform whole-cell patch-clamp recordings in monkey brain neurons *in vivo*. Whole-cell or intracellular membrane potential measurements have been just reported very recently in the cortex of awake monkeys through the use of blind patching procedure^31^ or the use of sharp electrodes^32^. As previously developed in the mouse cortex and cerebellum^23^, the targeted patching allows for the effective control and recording of the membrane potentials or a rapid delivery of fluorescent indicator/plasmid DNA/pharmacological agent into visually identified neurons in the intact primate brain. Using this procedure, one can also easily obtain the morphological information of the recorded cells according to *in vivo* two-photon imaging-based 3D reconstruction. In the present study, we show the use of targeted patching in marmoset monkey cortex for the recording and control of the intracellular dynamics of neurons *in vivo*. Under isoflurane anesthesia, we find the presence of slow oscillations of membrane potentials, namely UP and DOWN states in marmoset cortical neurons. This type of membrane potential activity was often found to be prevalent in cortical neurons during slow-wave sleep or under anesthesia according to the results from different species, e.g. rodents and cats^1, 19, 33^. However, owing to technical difficulty, in previous studies using extracellular recordings, these bistable membrane potentials of cortical neurons have been only inferred but not directly demonstrated to be existed in the marmosets^27, 34^. In addition, using whole-cell recordings, we have observed a stimulus intensity dependence of sensory-evoked intracellular responses in marmoset cortical neurons, which may provide a feasible tool for the study of cortical processing of sensory information at this level of resolution in nonhuman primate brain *in vivo*.

Using a combination of two-photon Ca^2+^ imaging and patch-clamp recordings, we have detected subthreshold synaptic Ca^2+^ transients in dendrites and spines in the marmoset cortical neurons. Intracellular pharmacological experiments reveal that these local synaptic Ca^2+^ transients are NMDA receptor-dependent. However, a recent study using an adeno-associated virus-based expression of GCaMP6 in the marmosets only reported global Ca^2+^ transients occurring throughout single dendritic branches^35^, which probably reflected backpropagation of APs and/or dendritic spiking, although no electrophysiological recordings were simultaneously performed. The failure of detection of synaptic input signals in their study could be due to the much larger movement artifacts caused by heartbeats or respiration activity than those in the mouse cortex^18^. In our study, we overcame this issue by adding a thick layer of agarose. In addition, our single cell labeling through the patch pipette yielded lower background fluorescence than that with virus-based labeling of genetically encoded calcium indicators^35^, which could significantly reduce the background contamination and thus improve the visibility of subthreshold dendritic signals. Therefore, our work extends the earlier study and can facilitate future investigations of mapping synaptic input signals in the neuronal dendrites in primate brain.

Since our imaging and electrophysiological recordings were done in one marmoset, this may raise concerns about the reliability, replicability and limitations of our approach. First, we obtained a total number of 10 whole-cell patched neurons from this animal. On average, no more than three attempts of inserting patch electrodes were needed to establish the whole-cell recording configuration. The recording time for each neuron was in the range of 30 minutes to 2 hours (two cells lasted up to ~2 hours and other eight cells varied from 30 minutes to 1 hour). The success rate of targeted whole-cell recording is not different from that was done in mice^1, 18^. However, experiments with this large animal involve some specific adaptations to achieve a successful recording. In particular, as compared to mice, we observed a slower and stronger respiration activity in marmosets (30–40 breaths per min in marmosets versus 90–120 breaths per min in mice)^18^, which produced much larger brain pulsations and thus became a major reason for the increased difficulty of targeted patching. This issue can be minimized by the use of an extra-thick layer of agarose (~3 mm), a large cranial window (~3 mm diameter), and, importantly, a very stable respiration control throughout the recordings by adjusting isoflurane at a relatively low level (0.6–0.8%). Second, dye filling using a patch electrode often suffers from dye leakage and insufficient filling of distant dendrites. Indeed, overall, only six out of ten recorded neurons had distant dendrites (defined as those at ~200 µm from their soma) clearly labelled with the Ca^2+^ dye. To minimize this problem, we restricted our dendritic analyses to the clearly visible dendrites of these neurons. In addition, to assure a sufficient dye filling, we restricted our series resistance of patch electrode to <30 MΩ and started dendritic imaging at least 20 min after formation of whole-cell configuration. Third, our approach provides the unique means to study single-cell behavior in detail without gaining much insight into the functions of the network it is connected to. Multicell bolus loading of synthetic calcium dyes^36^ or the use of virus-based labeling of genetically encoded calcium sensors^15^, together with single-cell patch-clamp recordings, may help to study single cells in the context of their network. Fourth, as compared to the use of GCaMP6s or other genetically encoded calcium indicators, our approach does not allow for recording the same neurons for days and weeks. Finally, this is a proof-of-principle report for the method that was performed in an anesthetized marmoset, which contains only limited amount of data. Based on these preliminary results, we did not observe any clear marmoset-specific properties. The future identification of any species-specific signature in the marmoset cortex requires more systematic investigations with delivering stimulation of different sensory features or under the conditions of higher order or complex behaviors.

We used the marmoset, as it has been recently received world-wide attention and is an excellent primate model for brain research due to its small brain size, high reproductive efficiency and availability of transgenic technologies^37, 38^. In particular, the smooth brain surface of marmosets affords huge advantages over other primate models for optical imaging and electrode penetration^7, 35, 39^. In addition, marmosets share many similarities in cognitive functions and social behavioral features with humans due to common descent^39, 40^. Therefore, functional recordings of the marmoset brain neurons using high-resolution technologies should shed light on the understanding of the human brain and also for the development of strategies for the diagnosis and therapy of psychiatric and neurological disorders^38, 41^. The procedure we demonstrated here would be a valuable addition to the toolbox for high-resolution functional recordings, particularly for acute experiments.

In conclusion, a combination of targeted patching and two-photon Ca^2+^ imaging of dendrites/dendritic spines will provide a useful tool to study single-neuron physiology, particularly how single neurons integrate sensory inputs through their dendrites^1, 3, 14^, in the entire primate brain networks under both healthy and diseased conditions. Although our recordings were done under anesthesia, this method can be possibly adapted to the awake primates in future.
